# Deciphering the binding behavior of flavonoids to the cyclin dependent kinase 6/cyclin D complex

**DOI:** 10.1371/journal.pone.0196651

**Published:** 2018-05-01

**Authors:** Jingxiao Zhang, Lilei Zhang, Yangcheng Xu, Shanshan Jiang, Yueyue Shao

**Affiliations:** 1 College of Chemistry and Environmental Engineering, Hubei University for Nationalities, Enshi, Hubei, China; 2 College of Chemistry and Chemical Engineering, Luoyang Normal University, Luoyang, Henan, China; Universita degli Studi di Torino, ITALY

## Abstract

Flavonoids, a class of natural compounds with variable phenolic structures, have been found to possess anti-cancer activities by modulating different enzymes and receptors like CDK6. To understand the binding behavior of flavonoids that inhibit the active CDK6, molecular dynamics (MD) simulations were performed on six inhibitors, chrysin (M01), fisetin (M03), galangin (M04), genistein (M05), quercetin (M06) and kaempferol (M07), complexed with CDK6/cyclin D. For all six flavonoids, the 3’-OH and 4’-OH of B-ring were found to be favorable for hydrogen bond formation, but the 3-OH on the C-ring and 5-OH on the A-ring were unfavorable, which were confirmed by the MD simulation results of the test molecule, 3’, 4’, 7-trihydroxyflavone (M15). The binding efficiencies of flavonoids against the CDK6/cyclin D complex were mainly through the electrostatic (especially the H-bond force) and vdW interactions with residues ILE19, VAL27, ALA41, GLU61, PHE98, GLN103, ASP163 and LEU152. The order of binding affinities of these flavonoids toward the CDK6/cyclin D was M03 > M01 > M07 > M15 > M06 > M05 > M04. It is anticipated that the binding features of flavonoid inhibitors studied in the present work may provide valuable insights for the development of CDK6 inhibitors.

## Introduction

Being a group of natural compounds with variable phenolic structures[1], flavonoids are ubiquitous in fruits, vegetables, tea and wine[2]. However, the daily intake of flavonoids is difficult to measure because of the diversity of dietary culture, and the structural complexity of flavonoids in various food sources. But in recent years there has been increased interest in investigating the pharmacological characters of flavonoids from food sources because of their versatile health benefits proved by various epidemiological studies[1]. As dietary components, flavonoids are deemed to exhibit health-promoting properties because of their high anti-oxidant activities in both *in vivo* and *in vitro* systems[3, 4]. The health benefits of flavonoids are supported by the abilities of those natural compounds to induce human protective enzyme systems, and abundant epidemiological studies suggest that the long-term consumption of diets rich in natural flavonoids offer protective effects against cancers, cardiovascular diseases, as well as bacterial and viral diseases[5]. Accordingly, researching mechanisms of action of flavonoids, such as their anti-cancer effects, is important to better understand their health benefits.

In recent years, flavonoids have been intensely investigated in the treatment of breast, cervical, prostate, ovarian and pancreatic cancers[6], and some of them, like quercetin, genistein and flavopiridol, have progressed to late stage trials for several oncological indications[7]. At the molecular level, flavonoids have been reported to modulate protein kinases, vascular endothelial growth factor receptors, epidermal growth factor receptors, platelet derived growth factor receptors and cyclin-dependent kinases (CDKs), which are all involved in cancer pathology[8]. Among them, CDKs, which are a group of serine/threonine kinases, have been extensively studied because of their essential roles in cell division cycle, transcription, differentiation, neuronal functions, as well as apoptosis[9, 10]. These kinases become active only in association with specific cyclin partner[9]. To date, at least 20 CDK family members and 30 cyclins have been reported[11, 12]. For example, CDK6 is activated by coexpression with D-type cyclins (like cyclin D1, D2, and D3)[13], and then drives cell division by phosphorylation of key proteins involved in the cell cycle progression, such as retinoblastoma protein (pRB) and pRB-related p107 and p130 proteins[6].

CDK6 plays a crucial role in the regulation of cell cycle progression. Up-regulation of CDK6 has been shown to be related to the development of several types of human cancers, such as breast, colon, pancreatic, bladder and oral cancers [14–17]. Although CDK6 is overexpressed at a very high frequency in cancer cells[17, 18], it has a low detectable level in healthy cells. These discoveries indicate a specific oncogenic role of CDK6 in cancer therapy, which may provide useful information to design the potent anti-cancer drugs with low toxicity[17]. Therefore, CDK6 is considered as a promising target for anti-cancer treatment.

Nowadays, several CDK6 inhibitors have been discovered, such as ribociclib (LEE011)[19], palbociclib (PD0332991)[20], abemaciclib (LY2835219)[21], AMG925[22], 7X[17], PD0183812[23] and flavonoid derivatives (like apigenin, fisetin, chrysin)[24]. Among them, palbociclib, ribociclib and abemaciclib are currently undergoing clinical investigation, AMG925, 7X and PD0183812 are in the preclinical stage of drug development[17]. These inhibitors compete with ATP and then bind to the ATP-competitive binding site, which result in the activity of the CDK6/cyclin D stopped. For example, flavonoid compound, fisetin has been reported to inhibit CDK6 with an IC_50_ value of 0.85 μM [24, 25]. In addition, molecular dynamic (MD) simulations were first applied in the research of three flavonoids (including fisetin, apigenin, and chrysin) as CDK6 inhibitors[26]. Despite of these work, the study on flavonoid derivatives as CDK6 inhibitors is limited. Therefore, in the present work, a series of flavonoid derivatives were selected to conduct comprehensive computational studies by a combination of docking, MD simulation, binding free energy calculation and weak interaction analysis. The models and information derived, we hope, may assist in understanding the binding behavior of flavonoids to CDK6/cyclin D complex.

## Materials and methods

### Preparation of ligands and protein

Flavonoids are based on a fifteen-carbon skeleton which consists two benzene rings linked via a heterocyclic pyrane ring[27]. They can be divided into several classes like flavones (e.g., luteolin, apigenin and chrysin), flavonols (e.g., quercetin, kaempferol, galangin and fisetin), flavanones (e.g., hesperetin, and naringenin), flavanonols (e.g., taxifolin), isoflavones (e.g., genistein and daidzein) and flavan-3-ols (e.g., catechin and epicatechin).

**Fig 1:**
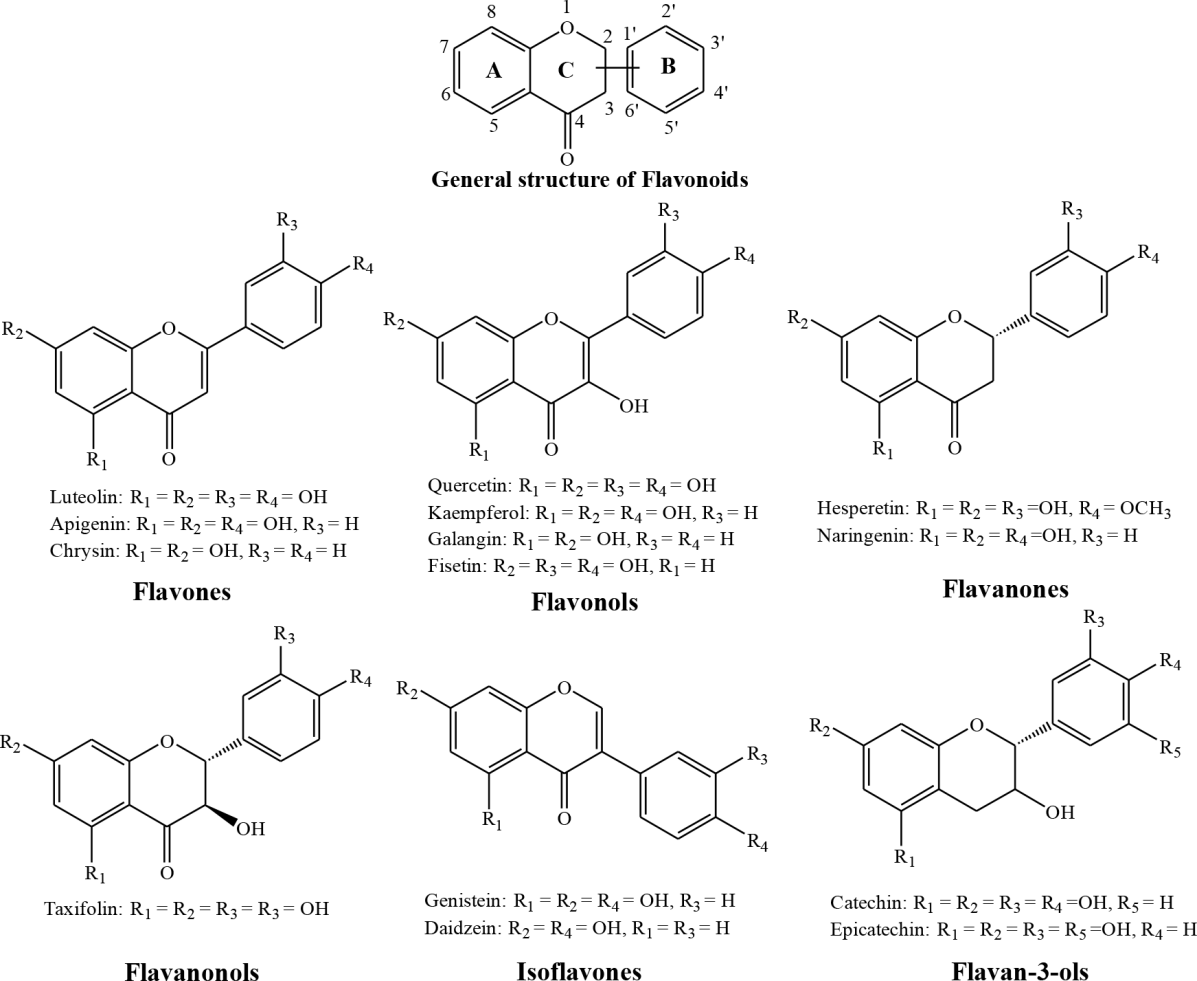
The major classes of flavonoids and the chemical structures of representative molecules that are discussed in this article.

The data regarding fisetin (as mentioned above) and the fact that natural flavonoids have been identified as protein kinase inhibitors for cancer chemoprevention would seem to point to these plant compounds as potential CDK6 inhibitors. However, only few flavonoids (like fisetin and chrysin) have been studied for understanding their binding modes on CDK6. Therefore, in this paper, all six categories, a total of 14 representative flavonoids (as displayed in Fig 1) are employed to explore their binding behavior on CDK6/cyclin D.

The initial structures of these flavonoids were downloaded from the NCBI PubChem database (https://pubchem.ncbi.nlm.nih.gov/)[28], and then subjected to full geometry optimization at a 6-31G(d,p)/B3LYP level of theory using the GAMESS package which uses frequency analysis to ensure the stability of molecular structure. The optimized structures of flavonoids were selected as initial conformations of molecular docking. Meanwhile, the CDK6/cyclin D in the active (DFG-in) conformation (PDB ID: 1XO2) was downloaded from the RCSB Protein Data Bank (http://www.rcsb.org/pdb/home/home.do) [29]. In preparation, the exogenous ligand and water molecules were removed, and the hydrogen atoms were added to the system.

### Molecular docking of flavonoids at the ATP-competitive binding site of CDK6/cyclin D

To reveal the binding affinity and investigate the interaction between flavonoids and CDK6/cyclin D, a docking operation was performed using AutoDock Vina program[30]. The software, which employs rapid gradient-optimization conformational search[31] to locate the most suitable binding site[32], can use all the rotatable bonds of the ligands to obtain a number of conformations from which the best binding mode could be achieved[33]. In the AutoDock Vina configuration files, the parameter *num_modes* was set to 8 and *exhaustiveness* to 10. A box of 40×40×30 Å^3^ around the binding site was built with a grid spacing of 1 Å, whose center was treated as the geometric center of the receptor. Finally, 14 flavonoids and the exogenous ligand of 3D structure (fisetin) were docked into the binding cavity of CDK6/cyclin D to find and select the lowest energy among 10 docking modes given by cluster analysis.

### Molecular dynamic simulation

To get the realistic binding modes, the frozen inhibitor-receptor complexes with the lowest binding affinities derived from the docking simulations were subjected to implement MD simulation with GROMACS 2016.3 software package[34]. The CHARMM27 all-atom force field was applied to describe the receptor, whereas general AMBER force field (GAFF) with AM1-BCC charges was performed to describe ligands. The simulation model was immersed in a periodic water box of cube shape. Prior to MD simulation, sodium ions were added to keep simulation system (which contains 85,629 atoms) at an electrically neutral state. The MD simulation was started by short (500 steps) energy minimization, followed by 100ps restricted dynamics to relax the solvent molecules. Solvent in the simulation systems was then equilibrated for 200ps while raising the temperature from 0K to 310K. Finally, a NPT simulation with a time step of 2fs was performed for 1000 steps[35]. During the process of simulation, the temperatures of protein-ligand complex and the remaining atoms in this system were controlled respectively by velocity-rescaling method. The long-range electrostatics were described with the PME (Particle Mesh Ewald)[36] algorithm, and the vdW (van der Waals) cutoff radii was set to 12Å.

### Binding free energy calculation

For each complex, 51 snapshot structures were extracted from the last 1ns along the MD trajectory at an interval of 20ps, and then were employed for binding free energy analysis. The MM-PBSA (molecular mechanics Poisson-Boltzmann surface area) method, which was implemented in g_mmpbsa tool[37, 38], was performed to compute the binding free energies of the inhibitors binding to CDK6/cyclin D complex. In this method, the binding free energy was calculated with the following equation[38]:

$$G_{binding}=G_{complex}-G_{protein}-G_{ligand}$$ (1)

where *G*_*binding*_ is the total free energy of the receptor-ligand complex, *G*_*protein*_ and *G*_*ligand*_ represent the total free energies of the separated receptor and ligand in solvent, respectively. The free energy for each separate entity was estimated by[38]:

$$G_{x}=E_{MM}+G_{solvation}$$ (2)

where x is the protein or ligand or complex. *E*_*MM*_ is the average molecular mechanics potential energy in vacuum, and *G*_*solvation*_ represents the free energy of solvation.

The vacuum potential energy, *E*_*MM*_, was calculated as following[38]:

$$E_{MM}=E_{bonded}+E_{non-bonded}=E_{bonded}+(E_{vdw}+E_{elec})$$ (3)

where, *E*_*bonded*_ is bonded interaction which contains bond, angle, dihedral and improper interactions, and *E*_*non-bonded*_ represents non-bonding interaction which includes van der Waals (*E*_*vdw*_) and electrostatic (*E*_*elec*_) interactions[38].

The solvation free energy (*G*_*solvate*_) was estimated as the sum of electrostatic solvation free energy (*G*_*polar*_) and apolar solvation free energy (*G*_*nonpolar*_):

$$G_{solvate}=G_{polar}+G_{nonpolar}$$ (4)

where *G*_*polar*_ was computed using the Poisson-Boltzmann (PB) equation. *G*_*nonpolar*_ was estimated by SASA (solvent-accessible surface area) method by the following equation:

$$G_{nonpolar}=\gamma S_{SASA}+\beta$$ (5)

where γ (equaled to 2.2 kJ·mol^-1^·nm^-2^) is a coefficient related to surface tension of the solvent, and β (equaled to 3.84 kJ·mol^-1^) is fitting parameter[38].

In this study, 20ns MD simulation was performed for equilibration and the last 1 ns of the trajectory was used for calculating the solvation free energy. The final calculated values were the average values of polar and apolar items of 51 conformations.

### Weak interaction analysis

The weak interaction analysis can be used to discover the noncovalent interactions between ligand and protein[39, 40], such as electrostatic, hydrogen bonding, steric repulsion and van der Waals forces[41]. The weak interaction on one frame of trajectory delivers the limited information of complex, but the average value of weak interactions based on the full trajectory of MD simulation can offer the more accuracy and smooth isosurfaces between inhibitor and CDK6[41, 42]. Therefore, in this paper, the protein-ligand complex after MD simulation in above section was treated as the original conformation. An additional MD simulation of 1ns duration was performed under the same condition except that all atomic coordinates of ligand were fixed, and 1001 frames of trajectories were extracted. The average reduced density gradient (aRDG)[43] was calculated based on averaged density gradient ($\bar{\nabla \rho (r)}$) and averaged density ($\bar{\rho (r)}$) of these 1001 frames by the following equation:

$$aRDG(r)=\frac{1}{2{\left(3\pi^{2}\right)}^{1/3}}\frac{\left\lfloor \bar{\nabla \rho (r)}\right\rfloor }{{\bar{\rho (r)}}^{4/3}}$$ (6)

The Multiwfn software[44] was employed to analyze the aRDG of protein-ligand complex. The number of grids was set to 150×150×150 in 3D spaces, and reduced density gradient (RDG) of each grid was calculated. The representation and color of aRDG was shown in VMD software[45].

### Experimental validation

To quantify the inhibitory activities of flavonoids on CDK6, the filter-binding assay was performed in 96-well filter plates. The total volume for each well was 0.1mL containing a final concentration of 20mM Tris-HCl (pH 7.4), 50mM NaCl, 1mM dithiothreitol, 10mM MgCl_2_, 25μM ATP (containing 0.25μCi of [γ-^32^P]ATP), 0.1μg of CDK6, 10μg of histone H1, and appropriate dilutions of inhibitor. The reaction was initiated by the addition of [γ-^32^P]ATP, and then incubated for 20min at 30°C. Samples were applied to P81 phosphocellulose papers strips (Whatman), and the strips were washed in phosphoric acid solution. The radioactivity was measured by liquid scintillation spectrometry[32]. IC_50_ values were calculated from the dose-response curves. All experiments were carried out in triplicate.

## Results and discussion

### Flavonoid-CDK6/cyclin D complexes

These 14 flavonoid inhibitors were separately docked into the ATP-competitive binding site of CDK6/cyclin D using AutoDock Vina software. The ligands in docked conformation and crystal structure (PDB ID: 1XO2) are well superimposed as displayed in supporting information S1 Fig, which verifies the reliability of the docking model. The lowest docked energy values were summarized in Table 1.

**Table 1:** The molecular docking results of flavonoids.

MOL ID	Name	Binding affinity (kcal/mol)	MOL ID	Name	Binding affinity (kcal/mol)
**M01**	Chrysin	-11.0	**M08**	Luteolin	-10.2
**M02**	Apigenin	-10.5	**M09**	Daidzein	-9.9
**M03**	Fisetin	-10.5	**M10**	Catechin	-8.5
**M04**	Galangin	-10.3	**M11**	Epicatechin	-8.6
**M05**	Genistein	-10.3	**M12**	Hesperetin	-8.3
**M06**	Quercetin	-10.4	**M13**	Naringenin	-8.7
**M07**	Kaempferol	-10.4	**M14**	Taxifolin	-8.4

As displayed in Table 1, all flavonoids bind to the active site with low binding energy values (from -8.4 to -11.0 kcal/mol). Additionally, it is worth noting that the order of binding affinities from the docking results is flavones (M01, M02) > flavonols (M03, M04, M06, M07) > isoflavones (M05, M09) > flavan-3-ols (M10, M11) > flavanones (M12, M13) > flavanonols (M14). On the basis of the docking results, flavones (M01, M02), flavonols (M03, M04, M06, M07) and isoflavones (M05), with the lower binding values (most negative), are selected as the candidate compounds for research, three of which (including M02, M03 and M01) have been studied by Khuntawee, W. in 2012[26]. Therefore, six focused flavonoids which contain M04, M06, M07, M05, M03 (the higher CDK6 inhibitor activity, being a reference), as well as M01 (the lowest value of binding energy, being a reference) are chosen as the final objects of this study.

### Stability of the simulated systems

**Fig 2:**
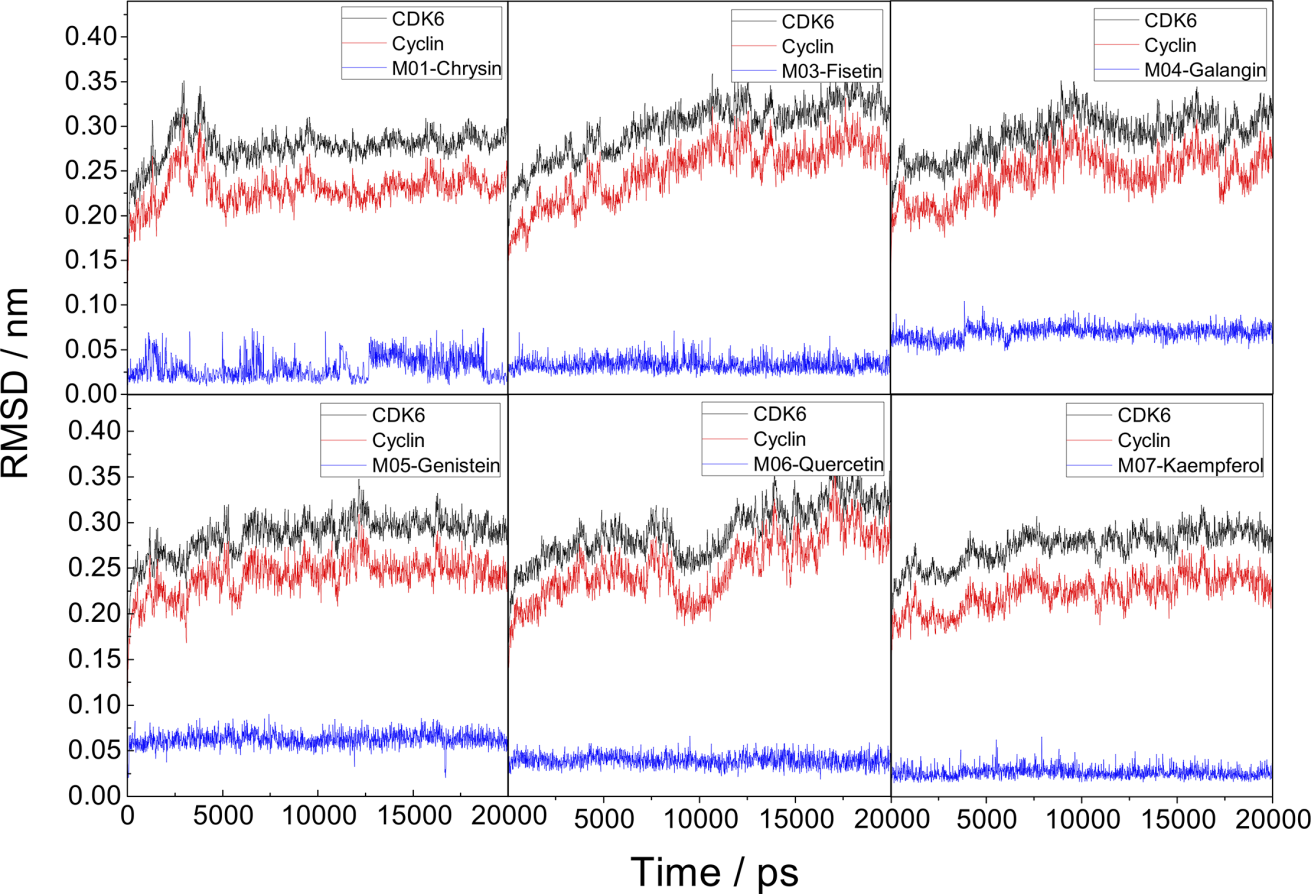
RMSD profiles of six simulated systems.

To investigate the stability of ligand-protein systems in aqueous solution, the docking conformations of six flavonoid-CDK6/cyclin D complexes, generated by AutoDock Vina, were taken as the initial conformations for MD simulation. The stability of these systems was determined by RMSD (root mean square distance) analysis of the MD trajectories, and the final results were plotted in Fig 2. The RMSDs for CDK6 (black), cyclin D (red), and inhibitors (blue) were displayed in this figure separately.

As displayed in Fig 2, the RMSD profiles of six systems are always less than 0.35nm, which suggests the suitability of MD simulation run, and providing a strong basis for further analysis. The RMSDs of CDK6 atoms are relatively higher than those of cyclin D atoms during the entire simulation. For the systems of CDK6/cyclin D with six flavonoids, the CDK6 is equilibrated with no obvious RMSD fluctuations after 5000 ps. The RMSD plots of six inhibitors tend to be steady along the simulation times. Compared to other three inhibitors, M03, M06 and M07 with small RMSD fluctuations (less than 0.05nm as displayed in Fig 2) may be more stable. To better understand the flexibility of protein in six systems, the MD trajectories from the last 15ns simulations were taken for further analysis.

### Binding pattern of inhibitors in CDK6/ cyclin D

**Fig 3:**
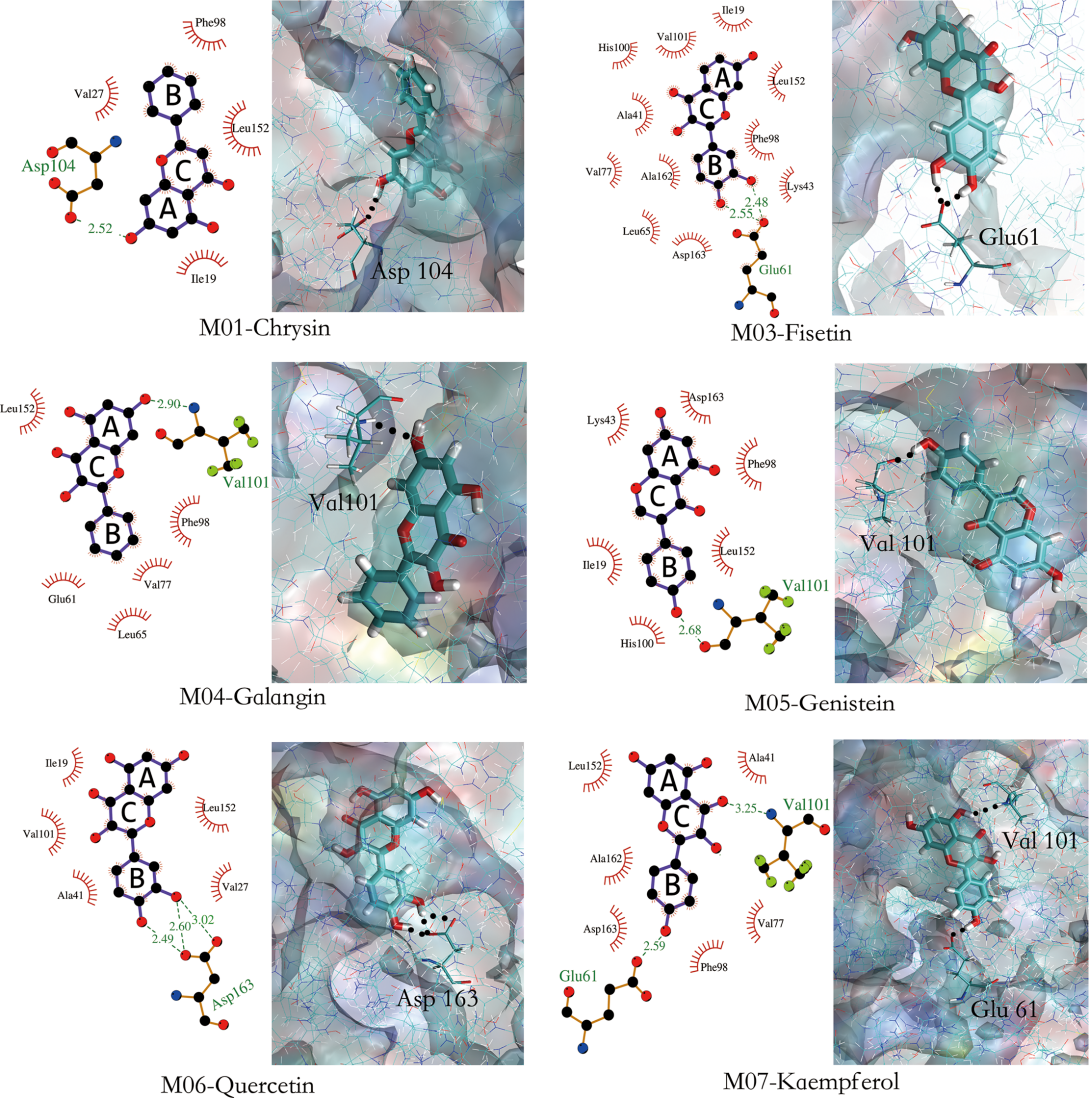
Flavonoid-CDK6/cyclin D interaction plots generated by LigPlot+[46] and the stereo view of flavonoid-CDK6/cyclin D interaction.

**Fig 4:**
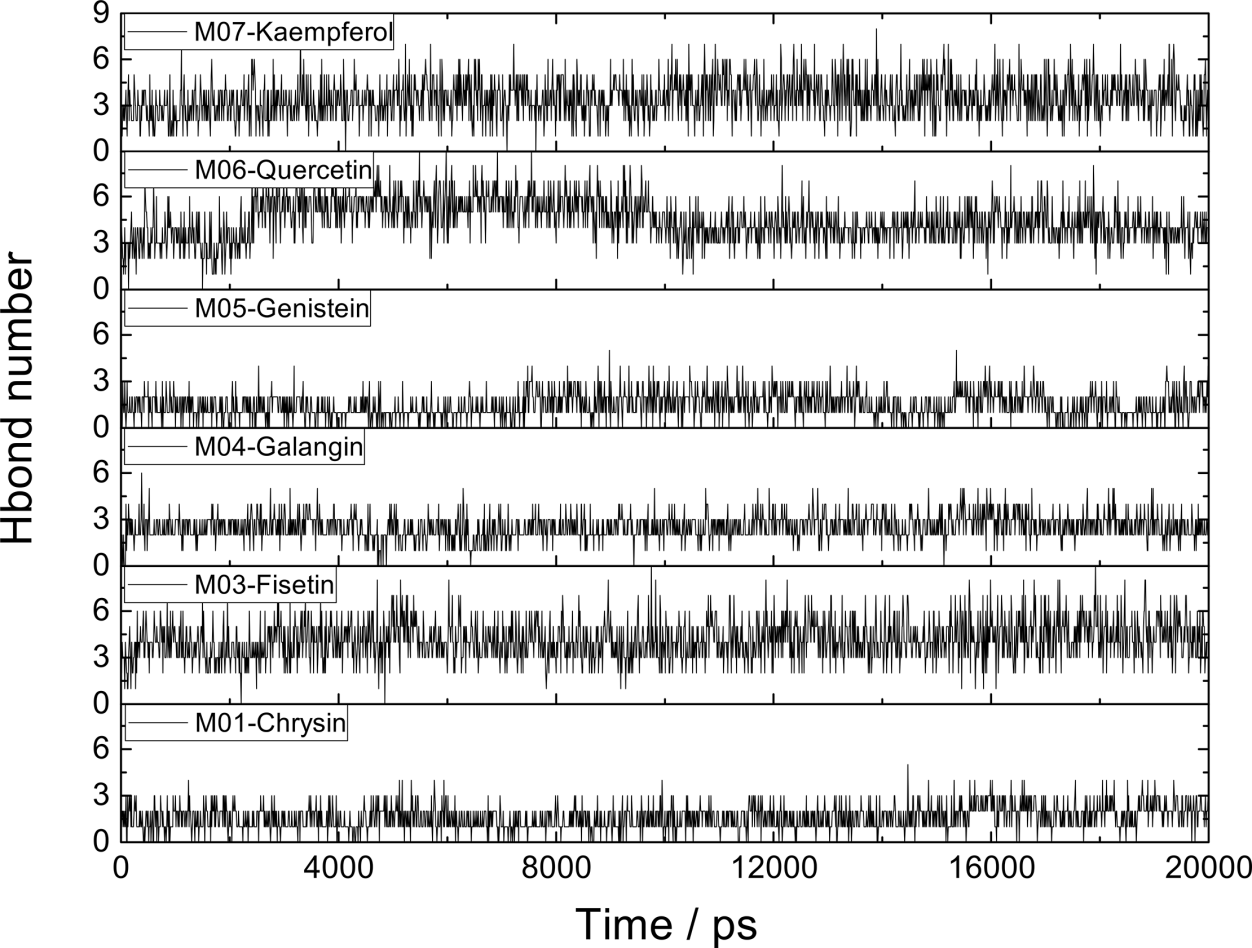
The number of hydrogen bonds formed between flavonoids and CDK6/cyclin D during the entire MD simulation.

All six inhibitors of this study are classified as flavonoids that contain a varying number of hydroxyl substitutions in three rings (ring A, B and C as shown in Fig 1). H-bond between hydroxyl substitutions as well as carbonyl group and the surrounding residues of CDK6/cyclin D is an important factor for flavonoids binding at the ATP-competitive site. To decipher such interactions, the number of hydrogen bonds of six inhibitors was calculated according to the following criteria: 1) donor−acceptor distance ≤ 3.5 Å; 2) bond angle ≥ 150°; 3) H-bond occupation ≥ 50%, and the results were summarized in Figs 3 and 4.

As shown in Figs 3 and 4 [46], protein-inhibitor interactions in term of the number of H-bonds in six complexes were found to be in the following order: M03-CDK6/cyclin D, M06-CDK6/cyclin D > M07-CDK6/cyclin D > M05-CDK6/cyclin D, M04-CDK6/cyclin D and M01-CDK6/cyclin D. To further explore the binding pattern of flavonoids to CDK6/ cyclin D complex, the similarity and difference were acquired by comparison of the H-bond features of six complexes. The main findings are summarized as follows:

1. The strong H-bonds are formed between the CDK6 and B-ring of the inhibitor, and the hydroxyl groups in 3’- and 4’-positions of B-ring are favorable for forming H-bonds, which fits well with the MD simulation results. For example, M07 and M04 whose structures only differ in 4’-position of B-ring such that M07 has hydroxyl group in 4’-position, thus it has one more H-bond at this position than M04 has. Additionally, compounds M06 (two H-bonds) and M07 (one H-bond) also follow the same trend at 3’-position of B-ring.
2. No H-bond is detected in the 3-position of C-ring of all six complexes, which may indicate that this hydroxyl group may be unfavorable for the formation of H-bond. This can be corroborated by M06, in which the hydroxyl group is at the 3-position of C-ring, however H-bond occupation (6.30%) is low in this position.
3. The hydroxyl group at the 5-position of A-ring may be also unfavorable for the binding and inhibitory efficiency. The results of H-bond analysis show that five inhibitors (including M01, M04, M05, M06, M07) have hydroxyl group at this position, however the H-bond occupations of this area are low, such as M01 with H-bond occupation of 0.25% at the 5-position of A-ring, and M06 having H-bond occupations of 21.74% in this position. Factually, the analogue of M06, M03 (without hydroxyl group at the 5-position of A-ring) has been proved to possess higher activity (IC_50_ = 0.85 μM) than M06 (IC_50_ = 26 μM)[24].

Significantly, H-bonds formed between B-ring of flavonoid and CDK6 make the flavonoid oriented and anchored well, which results in the fact that the other side of the ligand, i.e. A-ring, has no H-bond network. For example, as displayed in Fig 3, compounds M05, M06 and M07 (with one or two hydroxyl groups of B-ring) have at least one H-bond at the position of B-ring, however, there is no H-bond formed between the A-ring of these three compounds and CDK6. On the contrary, compounds M01 and M04 (without hydroxyl group of B-ring) have no H-bond at the position of B-ring, but one H-bond is formed between A-ring and CDK6. Therefore, the molecule with hydroxyl group at the 5-position of A-ring (like M06) may generate steric hindrance or unfavorable electronic contact, which results in the less activity than the compound (M03) without hydroxyl group at this position as mentioned above.

**Fig 5:**
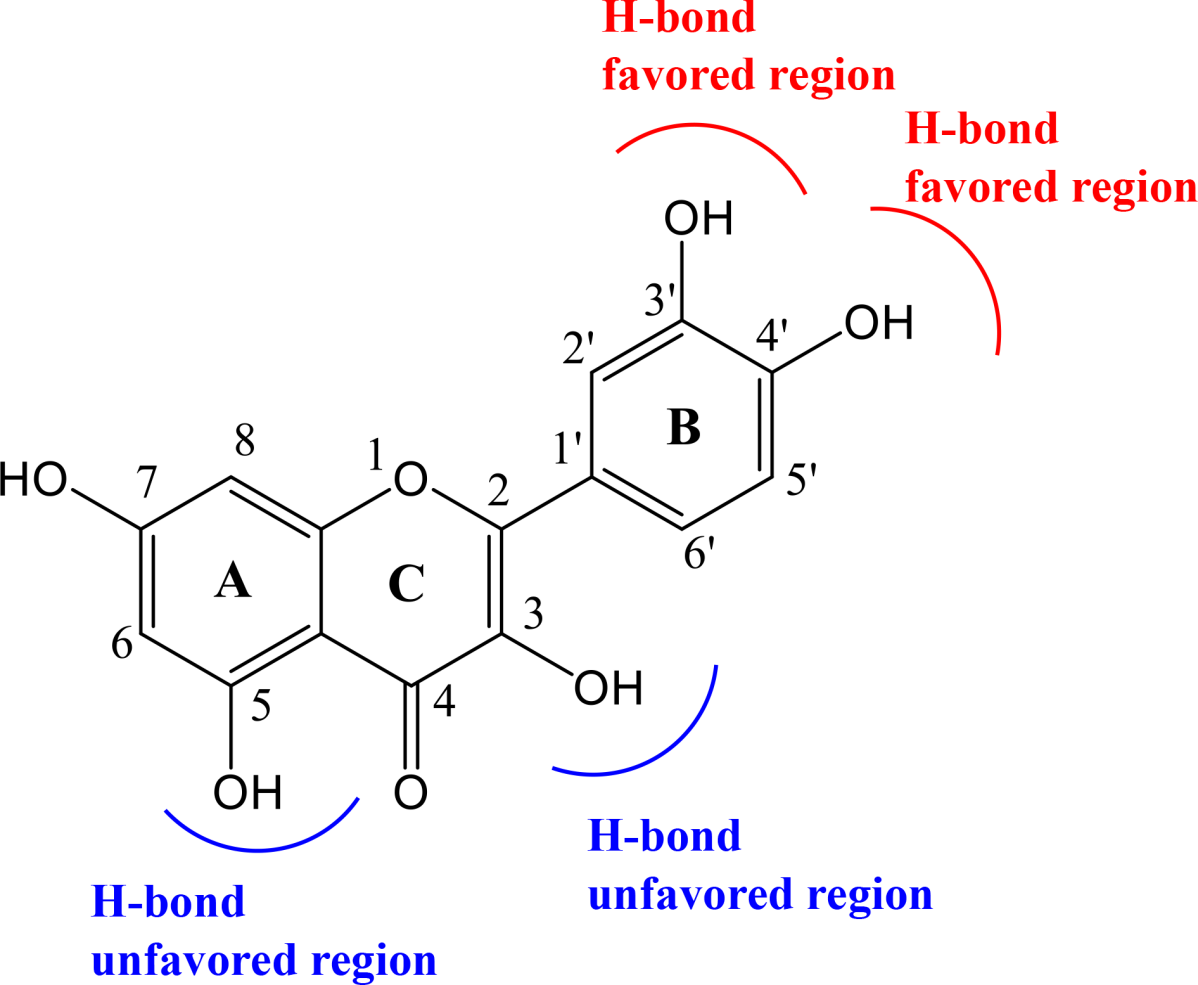
The H-bond features of flavonoid and CDK6/cyclin D.

**Fig 6:**
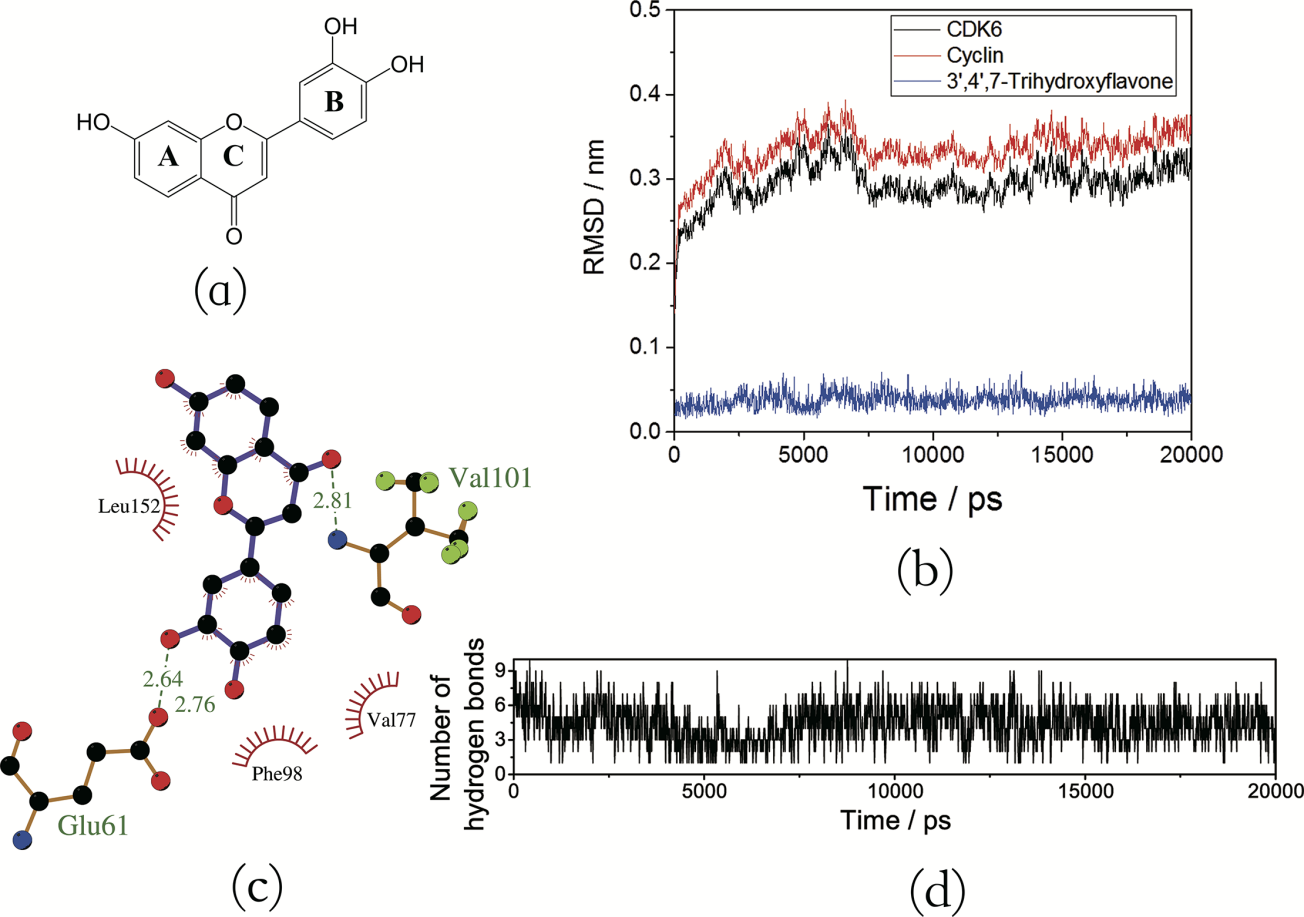
(a) Chemical structure of M15; (b) RMSD profile of M15-CDK6/cyclin D complex; (c) M15-CDK6/cyclin D interaction plot; (d) The number of hydrogen bonds during the MD simulation.

Fig 5 shows a graphical representation of the H-bond features between flavonoid (taking M06 as an example) and CDK6/cyclin D. To prove these conclusions, compound 3’, 4’, 7-trihydroxyflavone (M15) was used as test molecule for further research, because of the fact that it has hydroxyl groups at 3’- and 4’-positions of B-ring and does not exist hydroxyl group at the 3-position of C-ring and 5-position of A-ring (as shown in Fig 6A), which fits well with our findings. MD simulation for M15-CDK6/cyclin D complex was performed with the same conditions as previous, and the results were shown in Fig 6B, 6C and 6D.

Fig 6B depicts the trajectory of the RMSD of M15-CDK6/cyclin D complex during MD simulation. The results show that the RMSD value for the M15-CDK6/cyclin D complex rises to about 0.35nm in the first 6ns, and retains this value thereafter, which proves that the system behaves relatively stable after 6ns. In addition, small fluctuations were observed in the RMSD value of this test molecule (less than 0.05nm). Significantly, three strong H-bonds were detected in this system (as displayed in Fig 6C), and the interaction strengths vary from rather weak H-bonds in the M06-CDK6/ cyclin D system to relatively strong H-bonds in the M15-CDK6/cyclin D complex. For example, the H-bond from the 3’-OH group of M15 was found to have higher occupation (86.81%) than that of M06 (68.87%) in this position. In addition, during the entire simulation, the number of hydrogen bonds (about 3–7 bonds as shown in Fig 6D and supporting information S1 Table) formed between M15 and CDK6/cyclin D is always higher than other six systems. In summary, these simulation results of M15-CDK6/cyclin D prove the three findings as mentioned above.

### Binding free energy calculations

To probe the intermolecular binding motif of the flavonoids in the ATP-competitive site of CDK6, the MM-PBSA free energy calculation was employed using the g_mmpbsa tool for all seven flavonoid-CDK6/cyclin D systems. The binding free energy (Δ*G*_bind_) and the energy contributions were summarized in Table 2.

**Table 2:** Average MM-PBSA free energies (kcal/mol) of flavonoid-CDK6/cyclin D complexes.

MOL ID	Δ*E*_vdw_	Δ*E*_ele_	Δ*G*_sol_^polar^	Δ*G*_sol_^nonpolar^	Δ*G*_bind_
**M01**	-31.24±0.52	-32.85±0.43	40.64±0.32	-3.56±0.02	-27.01±0.35
**M03**	-33.36±0.54	-29.79±0.45	38.82±0.40	-3.87±0.02	-28.19±0.45
**M04**	-30.35±0.40	-6.63±0.47	27.62±0.52	-3.02±0.02	-12.60±0.50
**M05**	-29.91±0.35	-17.45±0.26	33.07±0.32	-3.63±0.02	-17.94±0.35
**M06**	-31.47±0.46	-29.26±0.30	44.90±0.37	-3.71±0.02	-19.56±0.40
**M07**	-33.11±0.49	-14.76±0.42	27.14±0.31	-3.33±0.02	-24.09±0.36
**M15**	-27.29±0.54	-27.52±0.65	37.48±0.60	-3.45±0.02	-20.79±0.51

The calculated results show that the order of Δ*G*_bind_ is M03 (-28.19 kcal/mol) > M01 (-27.01 kcal/mol) > M07 (-24.09 kcal/mol) > M15 (-20.79 kcal/mol) > M06 (-19.56 kcal/mol) > M05 (-17.94 kcal/mol) > M04 (-12.60 kcal/mol), which is consistent with the order of the experimentally determined IC_50_ values as displayed in Table 3. It is worth noting that more H-bonds are formed in M15-CDK6/cyclin D complex than in other six systems (as shown in Figs 4 and 6), however the binding free energy value of M15 is smaller than that of M03, M01 and M07, that may be because of its low Δ*E*_vdw_ contribution.

**Table 3:** IC_50_ values for inhibitors of CDK6.

MOL ID	IC_50_ (μM)	MOL ID	IC_50_ (μM)
**M01**	4.5±0.4	**M06**	26.3±1.9
**M03**	0.9±0.1	**M07**	19.8±1.6
**M04**	52.0±2.8	**M15**	23.3±1.5
**M05**	38.0±2.1		

The binding free energy values clearly indicate that, in all seven inhibitor systems, the Δ*E*_vdw_ contributions are found with a similar value (from -27.29 to -33.11 kcal/mol), whereas there exists a big difference in Δ*E*_ele_ contribution. In detail, the order of the Δ*E*_ele_ contribution is M01-CDK6/cyclin D > M03-CDK6/cyclin D > M06-CDK6/cyclin D > M15-CDK6/cyclin D > M05-CDK6/cyclin D > M07-CDK6/cyclin D > M04-CDK6/cyclin D, in which the corresponding Δ*E*_ele_ values are -32.58, -29.79, -29.26, -27.52, -17.45, -16.33, -14.76 and -6.63 kcal/mol. These data are with a certain difference to the number and occupation of hydrogen bonds, which may be the reason that, in addition to the hydrogen bond as the strong intermolecular force, there also exist weak electrostatic force between ligand and residues of protein. Another noteworthy difference is the unfavorable Δ*G*_sol_^polar^ energy contribution, and these values, to a large extent, are determined by the desolvation energy. Even though the structures of flavonoids are similar and their interactions with CDK6/cyclin D are similar, Δ*G*_sol_^polar^ and Δ*E*_ele_ contributions explain the difference in their binding.

**Fig 7:**
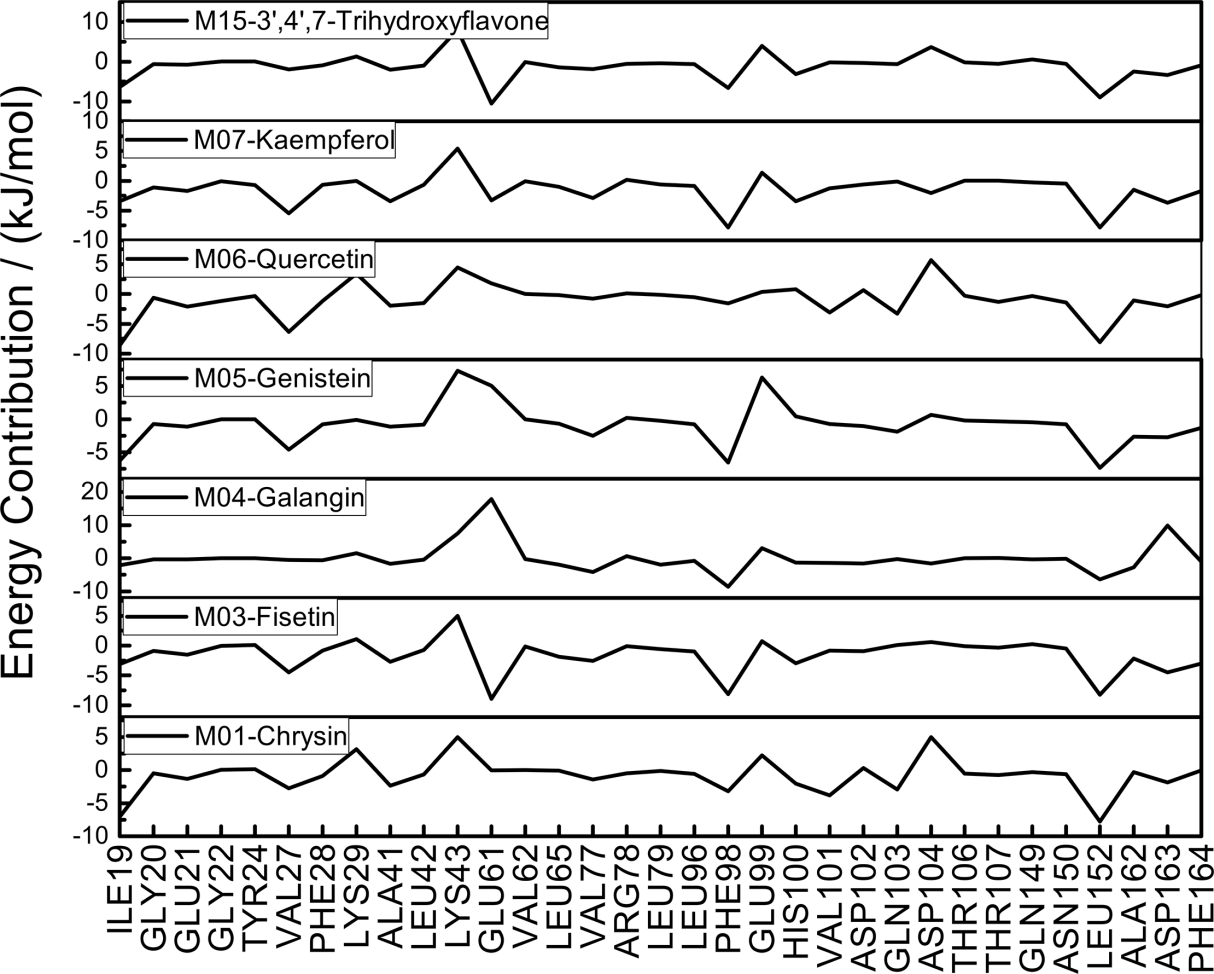
*Per* residue decomposition energies (Δ*G*bind^residue^) of crucial amino acids in various inhibitors.

To further analyzing the flavonoids-CDK6/cyclin D interactions, *per* residue decomposition approach was employed to quantify the role of identified crucial amino acids. The crucial amino acids contributing to the binding were protein residues which had intermolecular interactions with ligand, and the detailed results of *per* residue decomposition energy analysis for each flavonoid were displayed in Fig 7.

Previous results on H-bond studies of CDK6/cyclin D with seven flavonoids (as displayed in Figs 3 and 7) show that GLU61, ASP163, the hinge residue VAL101and the gatekeeper residue ASP104 are important for the formation of H-bonds, which is in accordance with the previous reports on CDK6/cyclin D as researched by W Khuntawee et al[26]. Analysis of *per* residue decomposition energies suggested that, compared with other residues as displayed in Fig 7, residues ILE19, VAL27, ALA41, GLU61, ASP163, LEU152, as well as two residues in the hinge region (including PHE98, GLN103) present lower values, and may play crucial roles in the binding of flavonoids. Taking LEU152 as an example, this residue provides the high degree of stabilization for all seven inhibitors, which is supported by the energy contribution of this amino acid (Δ*G*_bind_^residue^ ≤ -6.38 kJ/mol).

### Weak interaction analysis

**Fig 8:**
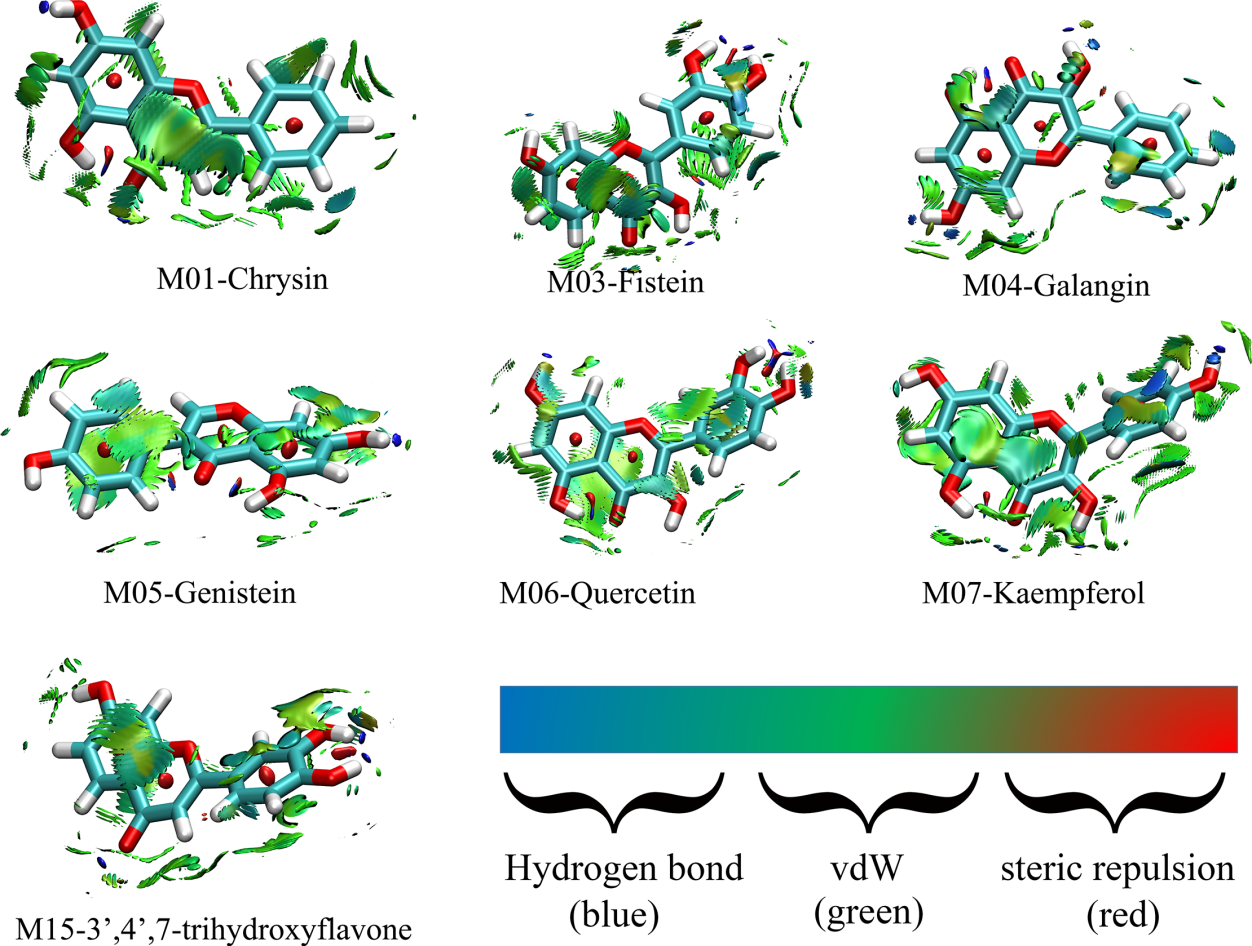
The weak interaction analysis in flavonoid-CDK6/cyclin D complex.

The weak interaction analysis can detail favorable and unfavorable interactions between receptor and ligand, and can also complement with H-bond analysis, steric repulsion and vdW interaction[41]. In this section, the weak interaction analysis is employed to profile the flavonoid-CDK6/cyclin D interaction mechanism, and the results are displayed in Fig 8.

As shown in Fig 8, the green and blue are the major colors on isosurfaces between the flavonoids and CDK6/cyclin D, which indicate that the vdW and H-bond interactions are the major factors in the binding efficiencies of flavonoids against the CDK6/cyclin D complex. In detail, among these seven systems, there are few differences according to the areas of green contours, thus there exists a small difference in Δ*E*_vdW_ contribution, which are in agreement with the findings of binding free energy calculations (as shown in Table 2). Additionally, several blue contours are found around the 4-position of the C-ring and 3’- and 4’-positions of B-ring respectively, which are also consistent with the data of MD simulation (as displayed in Figs 3 and 6).

## Conclusion

In this study, molecular docking, molecular dynamic simulation, binding free energy calculation and weak interaction analysis were applied to seek the detailed information on the binding mode of six flavonoid inhibitors (including M01, M03, M04, M05, M06 and M07) binding to the CDK6/cyclin D complex. The main findings are summarized as follows:

1. For 6 categories of flavonoids binding to CDK6/cyclin D complex, binding affinities in term of docking approach was found to be in the following order: flavones > flavonols > isoflavones > flavanones > flavanones > flavanonols.
2. The hydroxyl groups in 3’- and 4’-positions of B-ring of flavonoids were found to be favorable for forming H-bonds with CDK6/cyclin D, however the 3-OH on the C-ring and 5-OH on the A-ring were unfavorable, which were confirmed by the MD simulation results of the test molecule, M15, binding to CDK6/cyclin D at the ATP-binding pocket.
3. Both electrostatic (especially the H-bond force) and vdW interactions were found to be the important factors in the binding efficiencies of these flavonoids against the CDK6/cyclin D complex.
4. For CDK6/cyclin D complex, residues ILE19, VAL27, ALA41, GLU61, PHE98, GLN103, ASP163 and LEU152 may play crucial role in the binding of flavonoids.
5. On the basis of binding free energy calculation and experimental validation, the order of inhibitory affinities of flavonoids toward the CDK6/cyclin D complex was M03 > M01 > M07 > M15 > M06 > M05 > M04.

## Supporting information

S1 Fig Superposition of docked conformation (yellow) of co-crystal ligand over crystal structure (red). (TIF)

S1 Table The H-bonds formed between M16 and CDK6/cyclin D during the MD simulation. (PDF)
