# Supplementary figures and images for: Deciphering the binding behavior of flavonoids to the cyclin dependent kinase 6/cyclin D complex
Source: PLoS One. 2018 May 1;13(5):e0196651. doi: 10.1371/journal.pone.0196651 (PMC5929560; doi:10.1371/journal.pone.0196651)

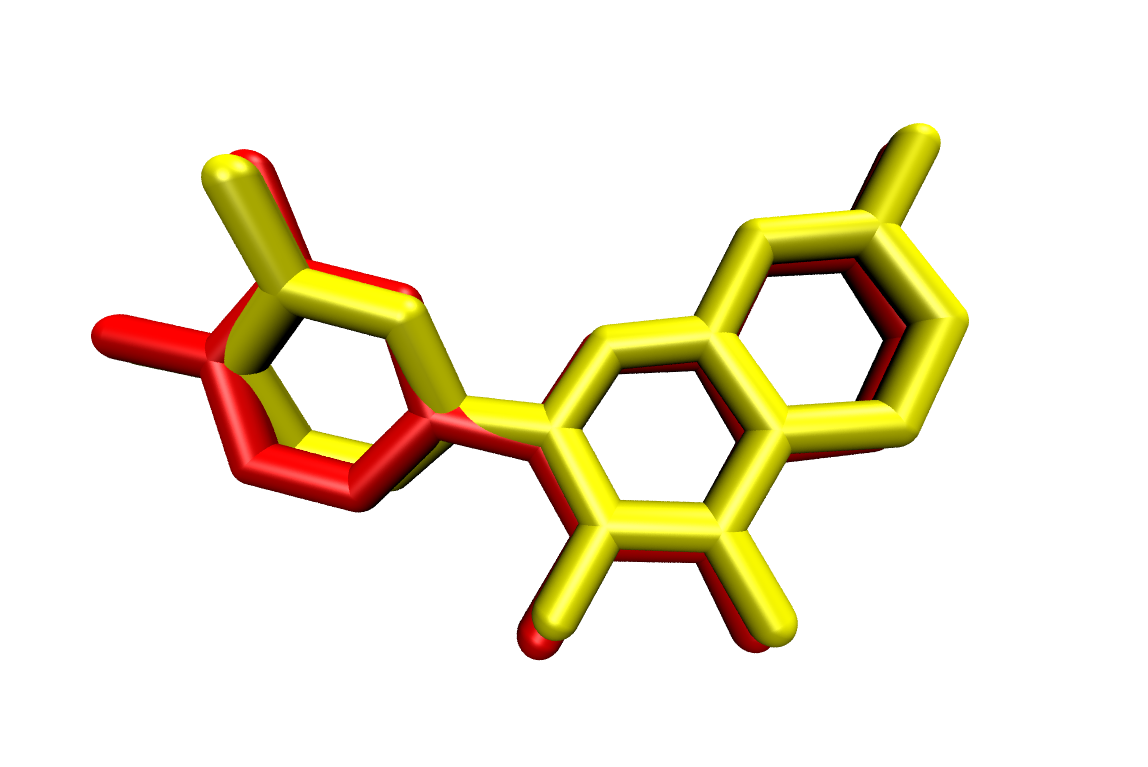

Supplement: S1 Fig — (TIF) [file pone.0196651.s001.tif]
