# Supplementary material for: Deciphering the binding behavior of flavonoids to the cyclin dependent kinase 6/cyclin D complex
Source: PLoS One. 2018 May 1;13(5):e0196651. doi: 10.1371/journal.pone.0196651 (PMC5929560; doi:10.1371/journal.pone.0196651)
Supplement: S1 Table — (PDF) [file pone.0196651.s002.pdf]

S1 Table. The H-bonds formed between M16 and CDK6/cyclin D during the MD simulation.

Donor	Acceptor	Occupancy	Donor	Acceptor	Occupancy
M15-Side-O20	GLU61-Side-OE2	88.76%	ILE19-Side-CD	M15-Side-C8	0.40%
M15-Side-O18	GLU61-Side-OE2	86.81%	M15-Side-O22	ILE19-Main-C	0.35%
VAL101-Main-N	M15-Side-O2	65.42%	LEU65-Side-CD2	M15-Side-O20	0.35%
M15-Side-O22	ASP104-Side-OD1	28.14%	ALA41-Side-CB	M15-Side-O2	0.30%
M15-Side-O20	GLU61-Side-CD	25.39%	LYS43-Side-NZ	M15-Side-C14	0.25%
M15-Side-O18	GLU61-Side-CD	13.29%	ASP163-Main-N	M15-Side-O18	0.25%
M15-Side-C8	VAL101-Main-O	11.69%	ASP163-Main-N	M15-Side-C14	0.20%
M15-Side-O22	ASP104-Side-OD2	9.05%	GLY20-Main-CA	M15-Side-O22	0.20%
ASP163-Main-N	M15-Side-C15	7.65%	M15-Side-C17	GLU99-Main-O	0.15%
HIS100-Main-CA	M15-Side-O2	6.40%	ASP104-Side-CB	M15-Side-O22	0.15%
M15-Side-O22	ILE19-Main-O	6.30%	ALA162-Side-CB	M15-Side-C17	0.15%
LYS43-Side-NZ	M15-Side-O18	5.75%	ASP163-Side-CB	M15-Side-O18	0.10%
M15-Side-O20	GLU61-Side-OE1	5.20%	VAL77-Side-CG2	M15-Side-C17	0.10%
M15-Side-O18	GLU61-Side-OE1	4.65%	LEU152-Side-CD1	M15-Side-C4	0.10%
M15-Side-O22	ASP104-Side-CG	4.15%	ILE19-Side-CD	M15-Side-C5	0.05%
ASP163-Main-N	M15-Side-O20	4.05%	LYS29-Side-CE	M15-Side-O2	0.05%
LEU65-Side-CD1	M15-Side-O20	3.40%	ILE19-Side-CD	M15-Side-C3	0.05%
M15-Side-C7	GLU99-Main-O	2.55%	LEU152-Side-CD1	M15-Side-C3	0.05%
GLN149-Side-NE2	M15-Side-O22	1.85%	VAL27-Side-CG1	M15-Side-C9	0.05%
ASP163-Main-CA	M15-Side-O18	1.40%	ILE19-Side-CD	M15-Side-C11	0.05%
HIS100-Side-CD2	M15-Side-O2	1.25%	M15-Side-O20	PHE98-Side-CZ	0.05%
ASP163-Main-N	M15-Side-C16	1.10%	LEU152-Side-CD1	M15-Side-O1	0.05%
VAL27-Side-CG1	M15-Side-O1	0.75%	ASP163-Main-CA	M15-Side-O20	0.05%
PHE164-Main-N	M15-Side-O20	0.55%			
